# Identification of a Splice Variant (c.5074+3A>C) of *BRCA1* by RNA Sequencing and TOPO Cloning

**DOI:** 10.3390/genes12060810

**Published:** 2021-05-26

**Authors:** Jinyoung Hong, Ji Hyun Kim, Se Hee Ahn, Hyunjung Gu, Suhwan Chang, Woochang Lee, Dae-Yeon Kim, Sail Chun, Won-Ki Min

**Affiliations:** 1 Department of Laboratory Medicine, Asan Medical Center, University of Ulsan College of Medicine, Seoul 05505, Korea; spi0430@gmail.com (J.H.); apoapo121@naver.com (J.H.K.); elisa970@naver.com (H.G.); sailchun@amc.seoul.kr (S.C.); wkmin@amc.seoul.kr (W.-K.M.); 2 Department of Biomedical Sciences, University of Ulsan College of Medicine, Seoul 05505, Korea; sayahn92@gmail.com (S.H.A.); suhwan.chang@amc.seoul.kr (S.C.); 3 Department of Obstetrics and Gynecology, Asan Medical Center, University of Ulsan College of Medicine, Seoul 05505, Korea; kimdy@amc.seoul.kr

**Keywords:** hereditary breast and ovarian cancer syndrome, *BRCA1* gene, RNA sequence analysis, cloning

## Abstract

Grading the pathogenicity of *BRCA*1/2 variants has great clinical importance in patient treatment as well as in the prevention and screening of hereditary breast and ovarian cancer (HBOC). For accurate evaluation, confirming the splicing effect of a possible splice site variant is crucial. We report a significant splicing variant (c.5074+3A>C) in *BRCA1* in a patient with recurrent ovarian cancer. Next-generation sequencing (NGS) of *BRCA1/2* from patient’s peripheral blood identified the variant, which was strongly suspected of being a splicing mutation based on in silico predictions. Direct RNA analysis yielded multiple transcripts, and TOPO cloning of the complementary DNA (cDNA) and Sanger sequencing revealed an aberrant transcript with an insertion of the first 153 bp of intron 17, and another transcript with the 153 bp insertion along with an exon 18 deletion. A premature termination codon was presumed to be formed by the 153 bp partial intron retention common to the two transcripts. Therefore, *BRCA1* c.5074+3A>C was classified as a likely pathogenic variant. Our findings show that active use of functional studies of variants suspected of altered splicing are of great help in classifying them.

## 1. Introduction

*BRCA1* and *BRCA2* are well-known tumor suppressor genes located on chromosomes 17 q21 and 13 q12 [1,2], respectively, and are involved in the repair of DNA double-stranded breaks [3]. Cancer susceptibility is increased when pathogenic mutations are present in these genes. Approximately 50% to 65% of women born with a deleterious mutation in *BRCA1* will develop breast cancer by age 70, and 35% to 46% will develop ovarian cancer by age 70. In the case of deleterious mutations in *BRCA2,* approximately 40% to 57% of women will develop breast cancer by age 70, and 13% to 23% will develop ovarian cancer by the same age [4,5]. Genetic testing and counseling are frequently offered to individuals suspected of having pathogenic mutations in *BRCA1/2;* examples are individuals with a family history of breast and/or ovarian cancer, patients with bilateral breast cancer, and patients with early onset of breast and/or ovarian cancer. Prevention and screening strategies for individuals harboring pathogenic *BRCA1/2* mutations are considered important [6,7]. Additionally, numerous clinical guidelines include *BRCA1/2* genetic testing in cancer work-up, and also suggest treatment options for breast and ovarian cancer patients carrying *BRCA1/2* mutations [8,9].

In this situation, grading the pathogenicity of *BRCA1/2* variants has great clinical importance. Genetic testing for *BRCA1/2* is performed extensively worldwide, and many resources and databases are currently available on *BRCA1/2* mutations and their clinical significance [10,11]. Still, many *BRCA1/2* variants continue to be classified as variants of uncertain significance (VUS) due to lack of supporting evidence. At the present time, 2860 of the 11561 *BRCA1* variants registered in ClinVar are classified as VUS [12]. In addition, novel variants are constantly being detected as more and more genetic testing is performed around the world.

Here, we describe a splice variant of *BRCA1* in a patient with ovarian cancer that was analyzed by a variety of molecular techniques including next-generation sequencing (NGS), Reverse transcription polymerase chain reaction (RT-PCR), Sanger sequencing, and TOPO cloning. This case demonstrates the process of obtaining functional evidence about a novel variant and classifying it in ways that can be clinically helpful.

## 2. Materials and Methods

### 2.1. DNA Extraction and Sequencing

To identify genetic variants of *BRCA1*/*BRCA2*, we extracted DNA from peripheral blood leukocytes using a QIAamp DSP DNA Blood Mini kit (Qiagen, Hilden, Germany) and prepared a library for NGS with a Customized Target Enrichment Kit (Dxome, Seongnam, Republic of Korea). We conducted NGS with a customized hybrid capture-based panel of *BRCA1* and *BRCA2* spanning all the coding regions and their flanking intronic regions (approximately 25 bp on either side), using a MiSeqDx V2 sequencing kit (Illumina, San Diego, CA, USA) on a Miseq Dx instrument (Illumina).

### 2.2. Bioinformatics

Raw sequencing BCL files were demultiplexed to make FASTQ files by the built-in MiseqDx software, Miseq Reporter (Illumina). Alignment to the reference sequence (GRCh37 assembly) was performed with BWA and Samtools. Post-alignment processing was performed with GATK MarkDuplicate, IndelRealigner, BaseRecalibrator and PrintReads. Variant calling was performed with GATK HaplotypeCaller and Varscan. Gene context information and dbNSFP data [13], including population frequency data from 1000 Genomes Project [14] and gnomAD [15] and prediction algorithm including ADA score [16] and RF score [17], were annotated with DxSeq software version 1.0.1 (Dxome).

### 2.3. RNA Analysis

To confirm the splice site change at the RNA level in the variant under study, we conducted RNA sequencing using primers developed in-house that target the affected region. RNA was extracted from the patient’s leukocytes with a High Pure RNA isolation kit (Roche, Indianapolis, IN, USA); the RNA was reverse transcribed with a RevertAid First Strand Synthesis kit (Thermo Fisher Scientific, Waltham, MA, USA) and the complementary DNA was Sanger sequenced with an in-house primer set using an ABI 3730 DNA analyzer (Thermo Fisher Scientific).

### 2.4. TOPO Cloning and Sequencing of the BRCA1 c.5074+3A>C Variant

A pair of primers was designed to amplify *BRCA1* gene fragments spanning variant c.5074+3A>C. The sequences were: gggtcaacaaaagaatgtcca, reverse: tcttccattgaccacatctcc. The PCR product was purified with a FavorPrep GEL/PCR Purification Kit (Favorgen, Ping-Tung, Taiwan) and cloned into TA vector using an RBC T&A Cloning Kit (Real Biotech Corporation, Taipei, Taiwan). The colonies obtained from ligation were screened by colony PCR using primers included in the RBC T&A Cloning kit, under conditions of (95 °C 3 min→30 cycle (95 °C 30 s→55 °C 40 s→72 °C 1 min)→72 °C 5 min). Positive clones yielding fragments of different sizes by PCR were cultured, and plasmid DNA was isolated with a miniprep kit (Exprep™ Plasmid SV mini, GeneAll, Seoul, Republic of Korea). The plasmid DNAs were analyzed by Sanger sequencing to obtain *BRCA1* sequences.

## 3. Case Description

**Figure 1:**
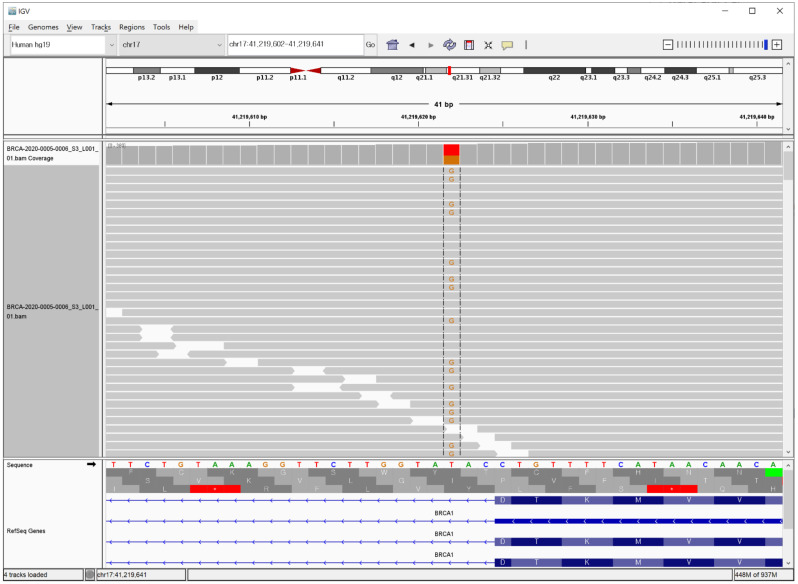
Identification of the *BRCA1* c.5074+3A>C variant by next-generation sequencing (NGS), visualized with Integrative Genome Viewer (IGV) (Reverse reading frame was shown).

A 53-year-old Korean woman, who had been previously diagnosed with stage 3 high grade serous ovarian cancer at age 51 and undergone chemotherapy with carboplatin and paclitaxel, visited a tertiary care hospital with ovarian cancer in relapse. The patient’s sister had a history of breast cancer, although her germline mutation status was not confirmed yet. NGS targeting the *BRCA1/2* gene region was conducted at a mean coverage depth of 544.4 x. Eleven variants were identified in *BRCA1,* and 12 in *BRCA2*. Apart from one variant in the intron of *BRCA1*, all the variants were predicted to be benign or likely benign based on the 2015 American College of Medical Genetics and Genomics and the Association for Molecular Pathology (ACMG/AMP) guidelines [18]. The identified unclassified variant was NM_007294.3(*BRCA1*): c.5074+3A>C; it was identified in the heterozygous state and found in 213 of 458 reads (Figure 1).

Another nucleotide change at the same position, (*BRCA1* c.5074+3A>C), has been reported to result in skipping of exon 17 or partial retention of intron 17, both of which are predicted to cause loss of normal protein function through protein truncation or nonsense-mediated mRNA decay [19], and therefore to be pathogenic. *BRCA1* c.5074+3A>C was found in ClinVar database (https://www.ncbi.nlm.nih.gov/clinvar/variation/867601, accessed on 8 April 2021), but not yet classified as pathogenic variant, due to intermediate function score produced by saturation genome editing assay [20]. The variant was not found in gnomAD [15]. Its ADA and RF scores were 0.9999 and 0.97, implying that it was damaging [21].

**Figure 2:**
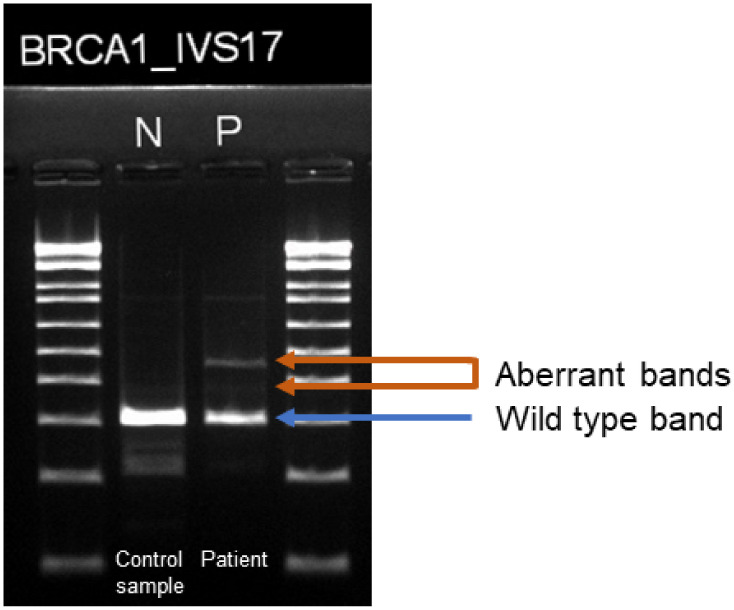
Electrophoresis of the RT-PCR product of RNA from the patient’s peripheral blood. Two faint bands (marked with orange arrows) can be seen in addition to the wild type band.

RNA analysis was performed to investigate the pathogenicity of the variant. However, when the cDNA produced by reverse transcription of RNA extracted from the patient’s peripheral blood was sequenced. However, the sequencing chromatogram was not interpretable. The presence of multiple transcript variants was also expected from the results of cDNA electrophoresis (Figure 2). To isolate multiple transcript variants and analyze their sequences, the cDNA was TOPO cloned.

**Figure 3:**
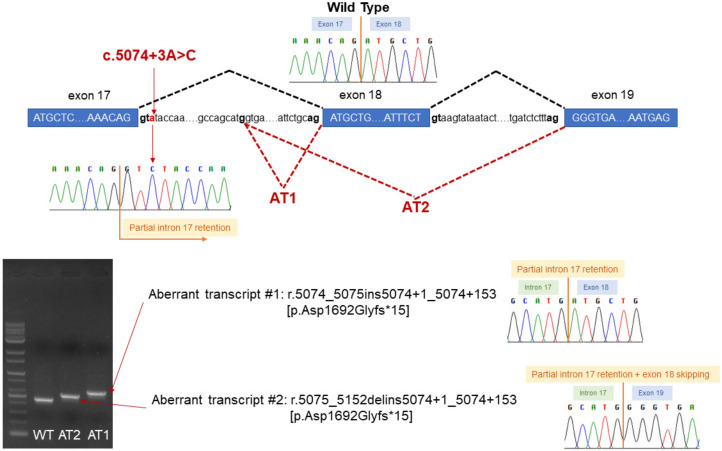
Scheme for the aberrant splicing caused by the *BRCA1* c.5074+3A>C variant, showing inactivation of the wild-type donor site of exon 17 and activation of a cryptic donor splice site.

By analyzing 23 colonies obtained by TOPO cloning, we identified 2 types of transcript in addition to the wild type. Two colonies showed an insertion of the first 153 bp of intron 17, and one colony showed the same insertion + exon 18 skipping (Figure 3). It was predicted that the presence of the 153 bp intron retention, which was common to the two non-wild type transcripts, would create a termination codon from the 15th codon and produce a truncated protein.

As described above, we have performed a functional study using RNA and cDNA derivatives of the *BRCA1* c.5074+3A>C variant, and obtained results suggesting that the variant is pathogenic. Therefore, the PS3 evidence level can be applied to it based on the ACMG/AMP guidelines. As mentioned above, PM2 was also applicable because it is not found in the population databases, and PP3 was applicable based on the results of in silico splicing prediction tools. By the application of one strong, one moderate, and one supporting piece of evidence, we were able to infer that *BRCA1* c.5074+3A>C is a likely pathogenic variant. 

## 4. Discussion

We have used this case to illustrate the process of classifying possible splicing variants as pathogenic using strong evidence obtained from functional studies (PS3). Splice site mutations are one important basis of *BRCA1* pathogenicity, and 226 of the 3403 likely pathogenic or pathogenic variants registered in ClinVar are splice site mutations [12].

*BRCA1/2* genetic testing is mainly performed at the DNA level. However, the splicing event takes place at the RNA level and cannot be directly observed by DNA sequencing. In most cases, effects on splicing are predicted and assessed using in silico algorithms that analyze DNA sequence data. Several splice site prediction software programs are available [22,23,24]. However, some use similar algorithms and may not yet have been validated, and their predictive capabilities can vary greatly from gene to gene. Therefore, in silico data is currently considered supporting evidence (PP3/BP4) and has a low evidence level [18], making it difficult to use as definitive evidence in variant classification. Additionally, pre-calculated databases such as dbscSNV are not helpful for unregistered variants. Therefore, a large numbers of possible splice site variants are classified as VUS and their clinical significances are not clearly provided, making additional intervention difficult.

According to the ACMG/AMP guidelines, direct analysis of RNA and/or complementary DNA derivatives is a useful functional approach that can provide a strong level of evidence (PS3/BS3) [18]. In the present case we attempted to confirm the splicing effect in vitro by sequencing the cDNA synthesized from RNA extracted from the patient’s peripheral blood. However, as shown in Figure 2, it appeared that the cDNA consisted of multiple transcripts and the products of sequencing could not be clearly read, so that the individual transcripts had to be isolated and sequenced. Usually, to extract the DNA separated via agarose gel electrophoresis, the band of interest is cut out from the gel and the DNA is extracted from the slice [25]. This procedure can be easily implemented even at the clinical laboratory level. However, in the case of mixtures of more than two transcripts, as in this case, it is difficult to clearly separate and sequence the individual transcripts by this method. In this study, TOPO cloning using TA vector was performed to overcome this limitation. As a result, colonies harboring wild type, aberrant transcript no. 1 and aberrant transcript no. 2 were successfully obtained, separated, and sequenced. TOPO cloning, though difficult to perform on a daily basis in a clinical laboratory, is a useful technique that can be used to determine whether a variant that produces multiple transcripts is a splicing mutation.

Although the ACMG/AMP guidelines assigns a strong level of evidence to functional studies, this is limited to well-established studies. Since not all functional studies are effective, it is recommended that they be interpreted carefully [18]. The Clinical Genome Resource (ClinGen) Sequence Variant Interpretation (SVI) Working Group has published additional guidance about assessment of the clinical validity of functional studies and determination of the strength levels of functional evidence [26]. Assays such as the protein truncation test [27] and various cell-based assays [28] can be used as approaches to loss-of-function mutations. However, these assays can generate unreproducible products, and can be of low level of evidence as they can be susceptible to experimental conditions or variability in controls. RNA sequence analysis is relatively reproducible and is a useful functional study to determine the impact of possible splice site variants on the integrity of spliced mRNAs.

RNA sequence analysis cannot directly observe nonsense-mediated decay (NMD) or protein expression. However, most NMD efficiencies of premature termination codons (PTCs) can be explained by NMD rules, and this tendency is stronger for dosage-sensitive genes [29] such as *BRCA1*. Additionally, trends in previous reports on disease relevance of other truncating mutations can serve as useful information in assessing splicing variant causing PTCs. Since a large number of *BRCA1* truncating mutations were previously reported as oncogenic mutations, and most *BRCA1* mRNAs bearing premature termination codons (PTCs) are known to be degraded by nonsense-mediated decay [30], when splicing assays reveal the presence of PTCs in *BRCA1*, this evidence can be sufficient for PS3 or PVS1 according to the recommendations [26].

*BRCA1* mutations can be indicated for targeted therapy and have significant implications for the treatment and prevention of illness in patients and their families. Therefore, reducing VUS as much as possible, and making definite judgments, can greatly assist in determining the direction of treatment and prognosis of patients and unaffected carriers. As in the present instance, performing in vitro functional studies by various techniques such as RNA/cDNA analysis and cloning, strong evidence can be added to further confirm pathogenicity. The active use of in vitro functional studies for mutations that are strongly suspected of being splice site changes can be of great help.

## 5. Conclusions

We concluded that *BRCA1* c.5074+3A>C is a likely pathogenic variant through demonstration of its impact on splicing by RNA sequence analysis and TOPO cloning. As in this case, conducting in vitro functional studies for possible splicing variants can aid in variant classification.

## Data Availability

Not applicable.
